# Intrathecal chemotherapy combined with systemic therapy in patients with refractory leptomeningeal metastasis of non-small cell lung cancer: a retrospective study

**DOI:** 10.1186/s12885-023-10806-5

**Published:** 2023-04-11

**Authors:** Tao Zhou, Shaofeng Zhu, Qiang Xiong, Jiongli Gan, Jianping Wei, Jing Cai, Anwen Liu

**Affiliations:** 1 Department of Oncology, The Second Affiliated Hospital of Nanchang University, No.1 Minde Street, Nanchang, Jiangxi Province 330000 People’s Republic of China; 2 Jiangxi Key Laboratory of Clinical Translational Cancer Research, Nanchang, Jiangxi Province 330000 People’s Republic of China; 3 Radiation Induced Heart Damage Institute of Nanchang University, Nanchang, Jiangxi Province 330000 People’s Republic of China

**Keywords:** Non-small cell lung cancer, Leptomeningeal metastasis, Intrathecal chemotherapy, Pemetrexed, Methotrexate

## Abstract

**Background:**

Leptomeningeal metastasis (LM) is the most devastating complication of non-small cell lung cancer (NSCLC), and its incidence is increasing. There is currently no standard treatment for LM, and the efficacy of traditional intravenous drug treatment is low, making refractory LM a difficult problem. In this study, we evaluated the efficacy and safety of intrathecal chemotherapy (IC)-based regimens in patients with refractory LM.

**Methods:**

We retrospectively enrolled NSCLC patients with confirmed LM who received IC and systemic therapy at the Second Affiliated Hospital of Nanchang University from December 2017 to July 2022. We analysed overall survival (OS), intracranial progression-free survival (iPFS), clinical response, and safety in these patients.

**Results:**

A total of 41 patients were enrolled. The median number of IC treatments was seven (range: 2–22). Seven patients received intrathecal methotrexate, and 34 patients received intrathecal pemetrexed. Clinical manifestations related to LM improved after IC and systemic therapy in 28 (68.3%) patients. The median iPFS in the whole cohort was 8 months (95% confidence interval [CI]: 6.4–9.7 months), and the median OS was 10.1 months (95% CI: 6.8–13.4 months). Multivariate analysis of the 41 patients with LM using a Cox proportional risk model showed that bevacizumab was an independent prognostic factor in patients treated with combination therapy (p = 0.002; hazard ratio [HR] 0.240; 95% CI: 0.097–0.595). Poor ECOG performance status remained a significant predictor of poor prognosis for survival (p = 0.048; HR 2.560; 95% CI: 1.010–6.484). Myelosuppression was the major adverse event over all IC dose levels. There were 18 cases of myelosuppression, 15 cases of leukopenia, and nine cases of thrombocytopenia. Eleven patients had myelosuppression above grade 3, including four with thrombocytopenia and seven with leukopenia.

**Conclusions:**

Combination therapy based on IC had good curative effects, was safe to use, and was associated with prolonged survival in NSCLC patients with LM. The use of bevacizumab is a good prognostic factor for NSCLC LM patients with combination therapy.

**Supplementary Information:**

The online version contains supplementary material available at 10.1186/s12885-023-10806-5.

## Background

Leptomeningeal metastasis (LM) refers to the spread of malignant cells to the leptomeninges, subarachnoid space, and other cerebrospinal fluid (CSF) compartments [1]. LM is a devastating complication that occurs in 3–5% of patients with advanced non-small cell lung cancer (NSCLC) [2]. However, its incidence is higher in subgroups of patients with targetable molecular driver mutations, including 9.4% of patients with epidermal growth factor receptor (*EGFR*) mutations [3, 4]. The recent growing incidence of LM is likely due to both improved supportive care and prolonged overall survival (OS) associated with new molecular therapies for patients with targetable mutations, particularly *EGFR* and anaplastic lymphoma kinase (*ALK*) mutations [3]. Targeted therapy is the first choice for LM patients with targetable mutations, while chemotherapy is the first choice for patients with wild-type genotypes. However, most LMs develop acquired resistance to targeted drugs, and more than half of all NSCLC patients have no sensitizing gene mutations [5]. The prognosis of LM thus remains very poor.

LM can damage the cerebral hemispheres, cranial nerves, and spinal cord and associated roots, resulting in a progressive decline in the general state of the patient and rapid progression to death if not treated. The transport restrictions associated with the blood‒brain barrier make traditional treatments futile, contributing to the poor prognosis. The clinical presentation of LM may include cranial nerve deficits, cauda equina symptoms or signs, visual disturbances, diplopia, hearing loss, neurocognitive syndromes, and signs related to intracranial pressure in the later stages (headache, nausea/vomiting, gait difficulties, encephalopathy, and somnolence). These symptoms may greatly impair the quality of life of patients with LM [6, 7]. The poor prognosis and severe symptoms of LM indicate an urgent need for improved treatment options in these patients.

LM is usually treated by direct intrathecal injection of chemotherapeutic drugs, including methotrexate, cytarabine (including liposomal cytarabine), and thiotepa. A pooled analysis showed cytological, clinical, and radiographic response rates to intrathecal chemotherapy (IC) of 55% (53–60%; n = 49), 64% (53–79%; n = 58), and 53% (n = 32), respectively, and the re-evaluated median survival time from the onset of treatment (n = 50) was 6.0 months (95% confidence interval [CI], 5.2–6.8) [8]. Pemetrexed has also recently been used for IC, and a phase I/II clinical study showed that intrathecal pemetrexed (IP) was associated with good safety and longer survival [9]. However, reports on the use of intrathecal therapy in LM patients with NSCLC are currently lacking. We therefore conducted a single-centre retrospective study to evaluate the efficacy and safety of IC in patients with refractory NSCLC and LM.

## Methods

### Patients

**Fig. 1:**
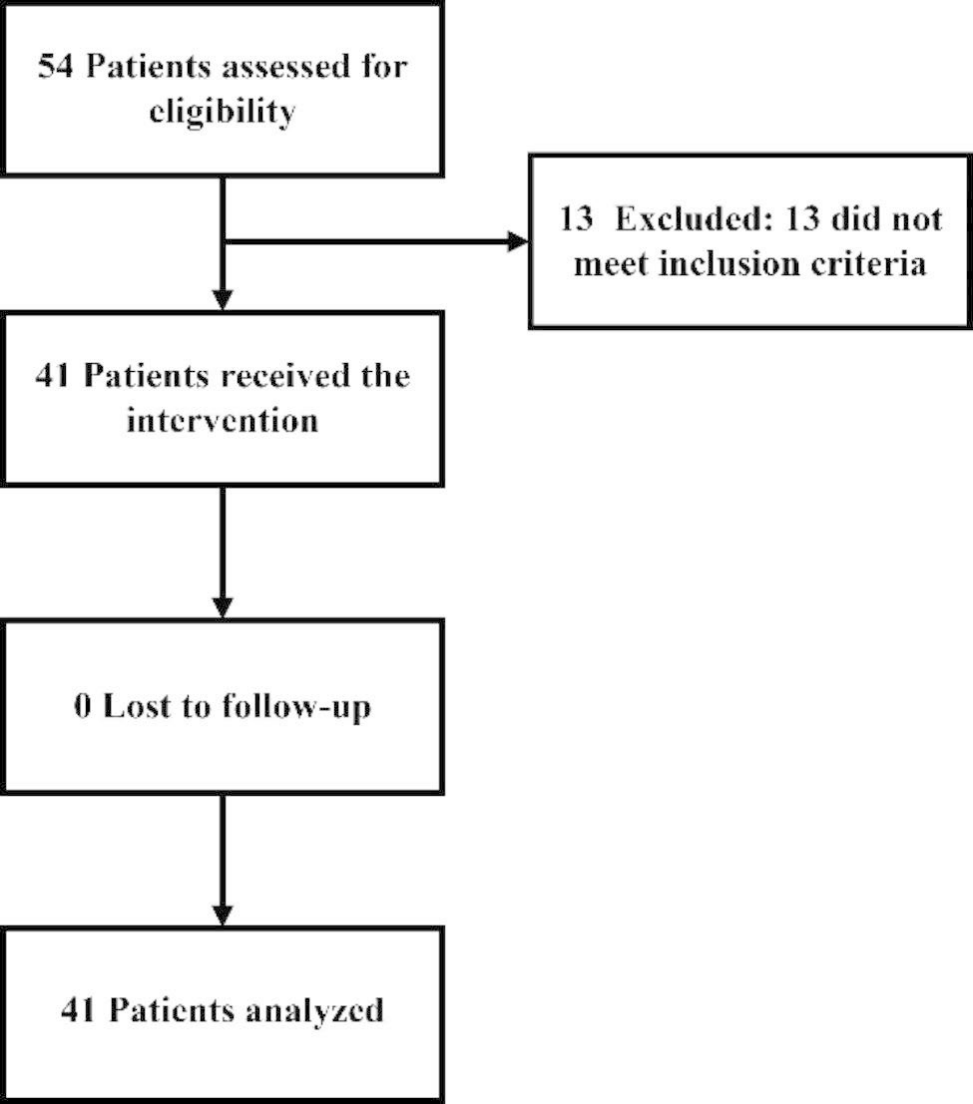
Consort diagram

A total of 41 patients treated at the Second Affiliated Hospital of Nanchang University were enrolled in this study from December 2017 to July 2022 (Fig. 1). LM was diagnosed according to the NCCN and European Association of Neuro-Oncology–European Society for Medical Oncology guidelines. We defined refractory LM as follows: (i) patients with actionable EGFR mutations diagnosed with LM after systemic therapy with TKIs or progression of known LM with TKI treatment; (ii) patients with EGFR Thr790Met (T790M) mutation, LM progression after failure of third-generation EGFR-TKIs; (iii) patients without EGFR T790M mutation, LM progression after failure of first-/second-/third-generation EGFR-TKIs; (iv) patients with LM progression after first- and second-generation EGFR-TKIs but refusal to undergo genetic retesting; and (v) wild-type patients with LM progression after failure of first-line therapy. The eligibility criteria were as follows: (i) patients were pathologically proven to have NSCLC; (ii) patients were diagnosed with LM positivity by cerebrospinal fluid (CSF) cytology (malignant cells) and/or typical findings (leptomeningeal enhancement or ventricle broadening) upon imaging; and (iii) patients received at least two doses of IC.

The exclusion criteria were as follows: (i) clinical manifestations of nervous system failure, including severe encephalopathy, grade III–IV white matter lesions confirmed by imaging examination, moderate or severe coma, or a Glasgow Coma Score less than nine points; (ii) refusal by patients or their family members to undergo invasive surgery; (iii) inability to cooperate with the lumbar puncture position; (iv) local infection at the lumbar puncture site; and (v) less than two intrathecal injections. This study was approved by the Research Ethics Committee of the Second Affiliated Hospital of Nanchang University.

### IC

The majority of patients underwent IC through lumbar puncture, while only three patients received IC through an Ommaya catheter. Pemetrexed or methotrexate was injected via lumbar puncture or Ommaya reservoir intrathecal injection twice per week in the first month, once per week in the second month, and then once every 4 weeks until the side effects became intolerable, the patient refused to continue the treatment, or the disease progressed. Methotrexate was administered at 12 mg per intrathecal injection. An initial dose of 10 mg of intrathecal pemetrexed was administered, followed by increments of 5 mg each time, increasing to a maximum dose of 50 mg, and the dose returned to the previous dose level when the patient developed grade 3 or higher myelosuppression or intolerable symptoms. Intrathecal dexamethasone was administered before and after intrathecal injection of the chemotherapeutic drugs (5 mg dexamethasone dissolved in 2 mL saline, 2.5 mg per dose). CSF pressure was measured before each intrathecal injection, and individualized systemic treatment and supportive treatment were given to each patient. All patients treated with intrathecal pemetrexed received supplemental folic acid (400 mg, once daily, orally) and vitamin B12 (1,000 mg, intramuscular injection 1–2 weeks before the first dose of pemetrexed and repeated every 9 weeks) throughout the treatment period to prevent side effects of pemetrexed. An informed consent form was signed before each IC treatment.

### Evaluation of responses and adverse events (AEs)

We assessed the patient’s condition by neurological symptom improvement and radiographic assessment with complete contrast-enhanced neuroaxis magnetic resonance imaging (MRI) according to the Response Assessment in Neuro-Oncology (RANO) Working Group on LM criteria. We did not use CSF changes in RANO to assess disease because the CSF method has low sensitivity; a negative result could reflect changes in the fluid and not necessarily reflect disease changes in the walls of the meninges and the cavity. AEs were assessed according to the National Cancer Institute Common Terminology Criteria for Adverse Events (version 4.03).

### Statistical analysis

Patient and treatment characteristics were summarized using descriptive statistics. Intracranial progression-free survival (iPFS) was defined as the time from IC treatment to LM progression or death. OS was defined as the time from IC treatment to death or follow-up. Survival analyses were performed using Kaplan–Meier estimates with 95% CIs. Statistical analyses of categorical variables were performed using Pearson’s χ^2^ test or Fisher’s exact test, as appropriate. Differences between groups were analysed by the log-rank test. The risk factors for OS were determined by univariate and multivariate Cox regression analyses. All statistical analyses were performed using SPSS software, version 27 (IBM Corp.), and a p value < 0.05 was defined as statistically significant. The final follow-up date was July 9, 2022.

## Results

### Baseline characteristics

A total of 41 patients underwent IC and systemic therapy at the Second Affiliated Hospital of Nanchang University from December 2017 to July 2022. All patients were diagnosed with lung adenocarcinoma. The median patient age was 56 years (range, 37–73 years). There were 17 males (41.5%) and 24 females (58.5%). Thirty-one patients (75.6%) were confirmed to have EGFR mutation, including one patient (2.4%) with *EGFR* mutation and *MET* amplification. At the start of intrathecal treatment, 22 patients (53.7%) had an Eastern Cooperative Oncology Group Performance Status (ECOG PS) score ≥ 2. The most common clinical presentations were dizziness (65.9%), headache (39%), limb weakness (17.1%), and altered mental status (14.6%). Forty patients had positive CSF cytology (at least one CSF examination during hospitalization). Thirty-two (78%) patients were MRI-positive. Concurrent brain metastases were reported in 32 (78%) patients at LM diagnosis. The clinical features and presentations of the patients are further listed in Tables 1 and 2.

**Table 1:** Basic characteristics of IP versus IM in the treatment of LM

Factor	IP	IM	All patients	P
No. of patients	34	7	41	
Age				0.923
<60	23	4	27(65.9%)	
≥60	11	3	14(34.1%)	
Sex				1.000
Male	14	3	17(41.5%)	
Female	20	4	24(58.5%)	
ECOG PS				0.302
< 2	17	2	19(46.3%)	
≥2	17	5	22(53.7%)	
CSF				0.376
Negative	0	0	0	
Positive	33	7	40(97.6%)	
Unknown	0	1	1(2.4%)	
MRI				0.299
Negative	9	0	9(22%)	
Positive	25	7	32(78%)	
Brain metastasis				0.299
Present	25	7	32(78%)	
Absent	9	0	9(22%)	
Systemic therapy before IC				0.372
1st/2nd-generation TKIs	22	2	24(58.5%)	
3rd-generation TKIs	18	3	21(51.2%)	
Chemotherapy	6	5	11(26.8%)	
Bevacizumab before IC				0.958
Yes	6	3	9(22%)	
No	28	4	32(78%)	
Gene mutation				0.334
EGFR	27	4	31(75.6%)	
MET	1	0	1(2.4%)	
None	7	3	10(24.4%)	

ECOG, Eastern Cooperative Oncology Group; PS, performance status; CSF, cerebrospinal fluid; MRI, magnetic resonance imaging; EGFR, epidermal growth factor receptor; MET, mesenchymal-to-epithelial transition

**Table 2:** Patients’ clinical manifestations (n = 41)

Clinical Manifestation	No. of patients (%)
Dizziness	27 (65.9%)
Headache	16 (39%)
Limb weakness	7 (17.1%)
Mental status changes	6 (14.6%)
Incontinence	2 (4.9%)
Epilepsy or convulsion	3 (4.6%)
Vision loss	2 (4.9%)
Hearing loss	1 (2.4%)

All patients had previous multiline treatment failure, including systemic chemotherapy, molecular targeted therapy, and antiangiogenic therapy. Prior to IC, 31 (75.6%) patients received EGFR-tyrosine kinase inhibitors (TKIs) with or without other agents (antiangiogenic or chemotherapy), 11 (26.8%) patients received systemic chemotherapy with or without other agents (immunotherapy or anti-angiogenesis), and one patient received both EGFR-TKI and c-Met tyrosine kinase (MET) inhibitors. Twenty-four of the patients treated with EGFR-TKIs received first-generation (gefitinib, erlotinib, or icotinib) and second-generation targeted agents (afatinib), 21 received third-generation EGFR-TKIs (osimertinib or almonertinib), and 14 patients received more than two EGFR-TKIs. Before IC, twenty-four patients had received a third-generation EGFR-TKI and LM progressed, or they received first- and second-generation EGFR-TKIs, but next-generation sequencing of cerebrospinal fluid indicated T790M negativity, and these patients were considered to have LM that had developed resistance to targeted therapy. Seven (17.1%) patients who had received only first-generation EGFR-TKIs refused to undergo next-generation sequencing of cerebrospinal fluid for personal reasons, so the sensitivity of subsequent targeted therapy was not determined. One patient had a MET amplification mutation detected by next-generation sequencing of cerebrospinal fluid. Nine (19.6%) patients received bevacizumab. Further treatment information is presented in Table 3.

### Treatment

Patients received a median of seven IC treatments (range: 2–22). Seven patients received a total of 38 intrathecal methotrexate (IM) injections (median: 5), and 34 patients received a total of 287 intrathecal pemetrexed injections (median: 8). The maximum dose of intrathecal methotrexate was 12 mg, and the maximum dose of intrathecal pemetrexed was 50 mg.

On the basis of intrathecal chemotherapy, systemic therapy including targeted therapy, systemic chemotherapy, immunotherapy, and antiangiogenic therapy were combined according to the ECOG PS and the clinical characteristics of the patients (Table 3). Twenty-six patients continued to receive targeted therapy, 14 received systemic chemotherapy, and one received immunotherapy. Among the patients who received targeted therapy, 25 patients received third-generation EGFR-TKIs (osimertinib or almonertinib), and one patient received second-generation EGFR-TKIs (afatinib). One patient had CSF genetic testing suggestive of *EGFR* 19 exon deletion and *MET* amplification and was treated with osimertinib in combination with savolitinib. The systemic regimen was unchanged in 12 patients, with the addition of intrathecal therapy. Twenty-six patients were treated with the antiangiogenic drug bevacizumab in combination with intrathecal treatment, including 19 patients who had not received bevacizumab before IC. Further treatment information is presented in Table 3 and Supplementary Table 1.

**Table 3:** Treatment

Patient	Gene mutation	CSF gene mutation before IC		Prior systemic treatment			Systemic treatment during IC	Neurological symptom assessment		MRI	Response determination	iPFS (m)	OS (m)
1	EGFR L858R	Unknown		Gefitinib, Osimertinib			TC + IM	Improved	Improved		Response	7.3	7.3
2	EGFR L858R	Unknown		Surgery, Icotinib, Almonertinib			Pemetrexed + IP	Stable	Stable		Stable	4	4
3	EGFR L858R	Unknown		Gefitinib, Erlotinib, Osimertinib			Osimertinib + IP + BEV	Stable	Stable		Stable	2.1	2.1
4	EGFR 19Del/T790M	Unknown		Gefitinib, Osimertinib			Osimertinib + IP	Improved	Stable		Stable	8.5	8.5
5	EGFR G719A	Unknown		Erlotinib			TC + IP	Worse	Not review		PD	2	2
6	EGFR 20Ins	Unknown		Osimertinib			TC + IM	Stable	Stable		Stable	3.5	3.5
7	Wild-type	Unknown		Surgery, DP, WBRT			TC + IM + BEV	Improved	Improved		Response	6.8	6.8
8	Wild-type	Unknown		PC + BEV			TC + IM + BEV	Improved	Stable		Stable	16.6	16.6
9	Wild-type	Unknown		PC + BEV			Pemetrexed + IM + BEV	Improved	Stable		Stable	11.6	24.3
10	Wild-type	Unknown		PC + BEV			Pemetrexed + IP + BEV	Stable	Stable		Stable	6.7	12
11	Wild-type	Unknown		PC + Pembrolizumab			Pemetrexed + IP	Worse	Not review		PD	2	2
12	Wild-type	Unknown		PC + BEV			PC + IP + BEV	Improved	Improved		Response	8.1	10.7
13	Wild-type	Unknown		Osimertinib + BEV			Osimertinib + IP + BEV	Improved	Improved		Response	15.1	17.8+
14	EGFR 19Del	Unknown		Gefitinib			Afatinib + IP + BEV	Improved	Stable		Stable	8.5	10.7
15	EGFR L858R	Unknown		Gefitinib			Osimertinib + IM + BEV	Improved	Improved		Response	10.1	10.1
16	EGFR L858R	Unknown		Gefitinib			Osimertinib + IP + BEV	Improved	Improved		Response	11.4	11.4
17	EGFR L858R	EGFR L858R		Gefitinib			Almonertinib + IP + BEV	Improved	Improved		Response	7	7
18	EGFR 19Del	EGFR 19Del, MET amplification		Surgery, Gefitinib			Osimertinib + Savolitinib + IP	Improved	Improved		Response	22.1+	22.1+
19	EGFR 19Del	EGFR 19Del		Surgery, Gefitinib			Osimertinib + IP + BEV	Improved	Stable		Stable	8	9.6
20	EGFR L858R	Unknown		Osimertinib, Pemetrexed + BEV			Osimertinib + IM + BEV	Improved	Improved		Response	3.6	13
21	EGFR 19Del	Unknown		Gefitinib, Osimertinib			Osimertinib + IP + BEV	Stable	Stable		Stable	14.5+	14.5+
22	EGFR L858R	Unknown		Icotinib + BEV			Osimertinib + IP	Improved	Stable		Stable	6	6.5
23	EGFR L858R	Unknown		Osimertinib			Osimertinib + IP	Improved	Improved		Response	14.7+	14.7+
24	EGFR 19Del	EGFR 19Del		Gefitinib, Osimertinib			Osimertinib + IP	Stable	Stable		Stable	6	7.3
25	EGFR L858R	Unknown		Icotinib, Osimertinib			Pemetrexed + IP + BEV	Improved	Improved		Response	5	9.8+
26	EGFR L858R	EGFR L858R		Surgery, Gefitinib, Almonertinib			Almonertinib + IP + BEV	Improved	Stable		Stable	5	6.4
27	Wild-type	Unknown		PC + Sintilimab			Sintilimab + IP + BEV	Improved	Improved		Response	6.6+	6.6+
28	EGFR 19Del	Unknown		Surgery, Gefitinib, Osimertinib			Osimertinib + IP + BEV	Stable	Stable		Stable	9.2	11.9+
29	EGFR 20Ins	EGFR 20Ins		TC + BEV, PC Sintilimab, Anlotinib			Almonertinib + IP	Improved	Stable		Stable	5.8	5.8
30	EGFR L858R	EGFR 19Del		Gefitinib, Osimertinib			Osimertinib + IP	Stable	Stable		Stable	3.9	3.9
31	EGFR L858R	EGFR L858R		Osimertinib			Osimertinib(160 mg/day) + IP	Worse	Not review		PD	1.7	1.7
32	EGFR 19Del	Unknown		PC, Osimertinib			Osimertinib(160 mg/day) + IP + BEV	Improved	Improved		Response	9.6+	9.6+
33	EGFR L858R	Unknown		Gefitinib, Almonertinib + BEV			Almonertinib + IP + BEV	Improved	Stable		Stable	6.4+	6.4+
34	EGFR L858R	Unknown		Gefitinib			Osimertinib + IP	Improved	Stable		Stable	8.7	13.2
35	EGFR 19Del	EGFR 19Del		Surgery, Gefitinib, Osimertinib			Osimertinib + IP + BEV	Improved	Stable		Stable	7.6+	7.6+
36	Wild-type	Unknown		PC			Paclitaxel + BEV + IP	Improved	Stable		Stable	4.5+	4.5+
37	Wild-type	Unknown		No			PC + BEV + IP	Worse	Not review		PD	4	4
38	EGFR 19Del	EGFR 19Del		Almonertinib			Almonertinib + BEV + IP	Improved	Stable		Stable	4.1+	4.1+
39	EGFR L858R	Unknown		Icotinib			Almonertinib + BEV + IP	Improved	Stable		Stable	4.1+	4.1+
40	EGFR L858R	Unknown		Gefitinib, Osimertinib			Pemetrexed + BEV + IP	Stable	Stable		Stable	4.1+	4.1+
41	EGFR L861Q	Unknown		Afatinib, Anlotinib Osimertinib			Osimertinib(160 mg/day) + IP	Improved	Stable		Stable	5	5.9

EGFR, epidermal growth factor receptor; ALK, anaplastic lymphoma kinase; PFS, progression-free survival; OS, overall survival; PC, pemetrexed and carboplatin; TC, paclitaxel and carboplatin; IP, intrathecal pemetrexed; IM, intrathecal methotrexate; BEV, bevacizumab; PD, progressive disease.

### Outcomes

**Fig. 2:**
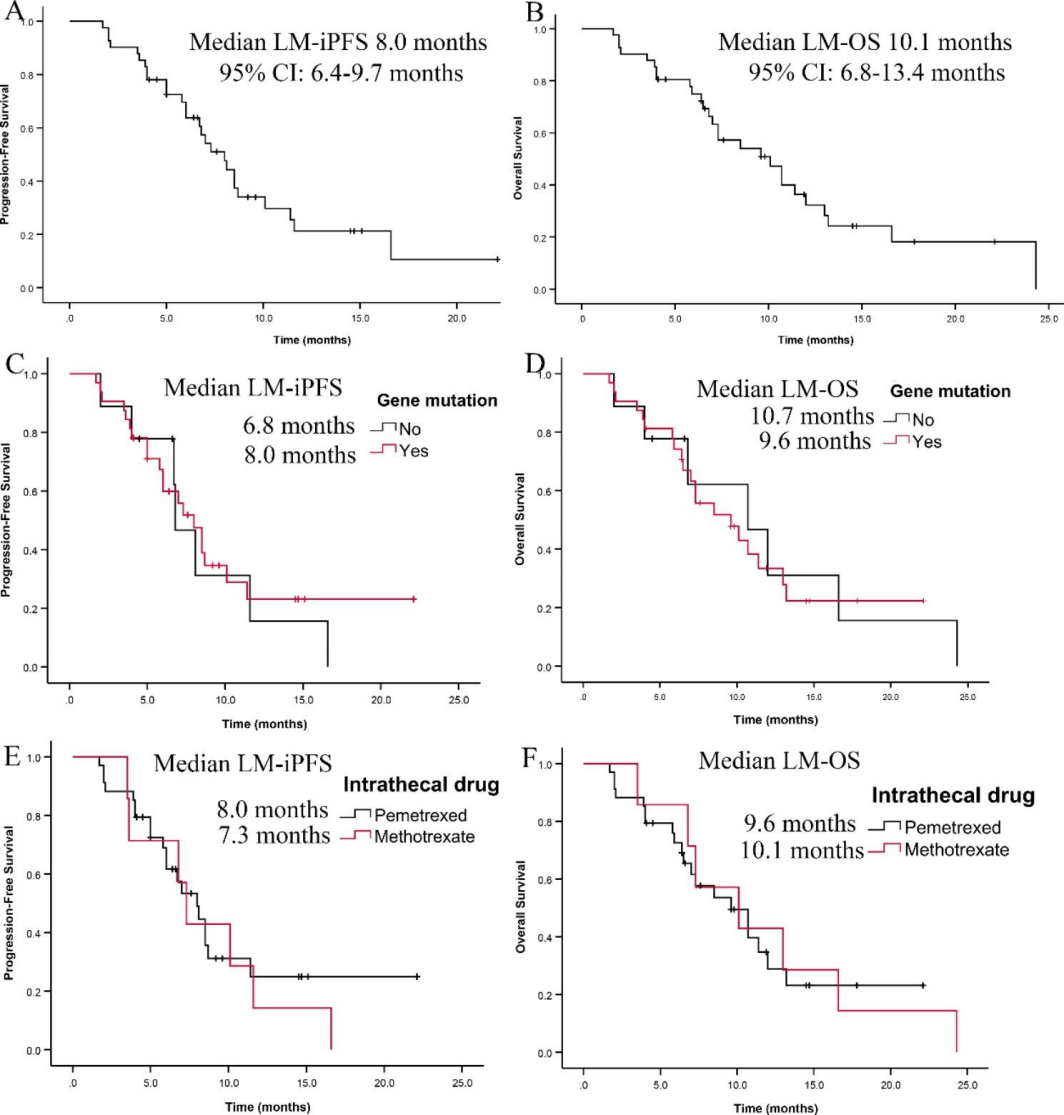
Kaplan‒Meier curve for leptomeningeal metastasis. (A, C, E) Progression-free survival (LM-iPFS); (B, D, F) overall survival (LM-OS).

All patients were followed up until July 9, 2022 (median follow-up time: 7.5 months). Twenty-seven patients died during the follow-up period, and another 11 patients had not reached iPFS (maximum iPFS: 22.1 months). The median iPFS was 8 months (95% CI: 6.4–9.7 months), and the median OS was 10.1 months (95% CI: 6.8–13.4 months) (Fig. 2A, B). The clinical manifestations related to LM improved in 28 (68.3%) patients after IC and systemic therapy, remained stable in 9 (22%) patients, and worsened in four (9.7%) patients. In patients with wild-type genotypes, the median iPFS was 6.8 months (95% CI: 5.1–8.5), and the median OS was 10.7 months (95% CI: 4.5–16.9), compared with 8 months (95% CI: 6.3–9.7) and 9.6 months (95% CI: 5.5–13.7), respectively, in patients with gene mutations (Fig. 2C, D). The median iPFS in patients receiving intrathecal methotrexate was 7.3 months (95% CI: 6.0–8.6 months), and the median OS was 10.1 months (95% CI: 2.9–17.3 months) (Fig. 2E, F). The median iPFS in patients receiving intrathecal pemetrexed was 8 months (95% CI: 6.0–10.0 months), and the median OS was 9.6 months (95% CI: 6.1–13.1 months) (Fig. 2E, F).

Factors affecting the prognosis of LM in terms of OS were included in univariate analysis. Sex, age, PS score, brain metastasis, gene mutation, number of IC treatments, and high CSF protein levels were not significantly different between patients receiving methotrexate and pemetrexed (p > 0.05); however, there was a significant difference between groups treated with and without bevacizumab (p < 0.05). Different PS scores were also found to have significant differences (p < 0.05). Multivariate analysis of 41 patients with LM using a Cox proportional risk model showed that combined bevacizumab was an independent prognostic factor for IC (p = 0.002; HR 0.240; 95% CI: 0.097–0.595) and that poor ECOG performance status remained a significant predictor of poor prognosis for survival (p = 0.048; HR 2.560; 95% CI: 1.010–6.484) (Table 4).

**Table 4:** Cox proportional hazard model analysis of factors affecting patient overall survival (n = 41)

	Univariate Analysis			Multivariate Analysis		
Factors	p	HR	95% CI	p	HR	95% CI
Age (≥ 60 vs.<60 year)	0.279	0.649	0.297–1.419	0.745	—	—
Sex (female vs. male)	0.513	1.296	0.596–2.821	0.273	—	—
ECOG PS (<2 vs.≥2)	0.013*	2.862	1.246–6.575	0.048*	2.560	1.010–6.484
Brain metastasis (no vs. yes)	0.969	1.018	0.405–2.562	0.644	—	—
Gene mutation (no vs. yes)^a^	0.840	1.100	0.437–2.765	0.739	—	—
Combined with BEV (no vs. yes)	0.022*	0.404	0.186–0.877	0.002*	0.240	0.097–0.595
CSF protein (Normal vs. Increased)	0.102	3.427	0.782–15.011	0.064	—	—
Number of IC	0.202	0.948	0.873–1.029	0.813	—	—

^a^ Gene mutations including sensitive gene mutations and rare mutations sensitive to targeted therapy.

*p<0.05

ECOG, Glasgow prognostic score; BEV, bevacizumab; CSF, cerebrospinal fluid; IC, Intrathecal chemotherapy

**Table 5:** AEs Systemic Chemotherapy Targeted Therapy

Toxicity	Gr 1	Gr 2	Gr 3	Gr 4	Gr 1	Gr 2	Gr 3	Gr 4
Haematologic toxicities	4	3	3	3	3	3	3	2
Leukopenia	3	2	3	1	1	2	2	1
Thrombocytopenia	1	1	0	2	2	1	1	1
EHA	1	0	0	0	0	0	0	0
Acute cerebral meningitis	0	0	0	0	0	0	0	0
Leukoencephalopathy	0	0	0	0	0	0	0	0

AEs, Adverse events; Gr, Grade; EHA, Elevation of hepatic aminotransferases

### Safety and AEs

No patients died of treatment-related side effects. The most common AE was myelosuppression, usually related to AEs of chemotherapy, targeted therapy, or IC. There were 18 cases of myelosuppression, 15 cases of leukopenia, and nine cases of thrombocytopenia. Eleven patients had myelosuppression above grade 3, including four with thrombocytopenia and seven with leukopenia. Thrombocytopenia and leukopenia occurred simultaneously in three patients. One patient had elevated liver transaminases. Adverse reactions of grade 3 or above occurred in 6 patients with systemic chemotherapy. Five patients with targeted therapy had adverse reactions above grade 3. All patients improved after symptomatic treatment, including recombinant human granulocyte colony-stimulating factor, recombinant human thrombopoietin, and hepatoprotective drugs such as glutathione. Further treatment-related side effect information is presented in Table 5 and Supplementary Fig. 1.

## Discussion

This retrospective study showed that multiple IC treatments resulted in good survival benefits and tolerable side effects in patients with LM related to NSCLC. The median iPFS was 8 months (95% CI: 6.4–9.7), and the median OS was 10.1 months (95% CI: 6.8–13.4) in treated patients. In addition, bevacizumab was identified as a significant independent prognostic factor in relation to combination therapy in LM patients (p = 0.002).

Although the treatment of lung cancer has developed rapidly, LM remains one of the most serious complications of NSCLC. IC is one of the few currently available treatment modalities that can deliver chemotherapeutic agents directly into the CSF without crossing the blood‒brain barrier. Increasing evidence suggests that IC has good efficacy in LM patients with NSCLC [9–11]. Dizziness (65.9%) and headache (39%) were the main clinical manifestations in the 41 patients in the current study, with serious impacts on quality of life. Systemic therapy combined with IC treatment relieved the clinical symptoms of LM, with improvements in 68.3% of patients [11]. The response rate of clinical symptoms was similar to previous retrospective studies, but the iPFS (8 months vs. 9.6 months) and OS (10.1 months vs. 20 months) were shorter in the current study [10, 11]. There were several possible reasons for this. First, there was a high proportion of patients without sensitizing gene mutations or resistance to targeted therapy in this study. Second, 12 patients (29.3%) did not change their systemic treatment plan after the progression of LM and only added IC. Finally, there was a high proportion of critically ill patients in this study, with 53.7% having an ECOG PS score > 2. Targeted therapy is currently updated rapidly in patients with sensitizing mutations, but systemic chemotherapy combined with IC remains the main treatment option for LM patients without such mutations. The iPFS and OS for the nine patients without sensitizing mutations who received systemic chemotherapy combined with IC were 6.8 months and 10.7 months, respectively, which were significantly longer than the 7.5 months in a previous pooled analysis [8]. However, the incidence of LM was lower in patients without sensitizing mutations, and we only included nine such patients. Further studies with larger sample sizes are therefore needed to verify these results.

Methotrexate and pemetrexed are currently the most commonly used intrathecal chemotherapeutic agents for NSCLC [12]. Pemetrexed is a cell cycle-specific antimetabolite folate inhibitor similar to methotrexate that has been used for systemic chemotherapy in patients with first- or second-line nonsquamous NSCLC and is thought to reduce the risk of death from brain metastases or LM [13, 14]. IP treatment has been shown to achieve high disease control and clinical response rates in patients already receiving intravenous pemetrexed chemotherapy [10]. Pemetrexed maintained high CSF concentrations for prolonged periods of time in a rat IP model [15]. In addition, phase 1/2 clinical trials of IP in patients with LM from NSCLC showed manageable toxicity and good efficacy [9]. Methotrexate is another intrathecal option for LM but is not specifically designed for patients with NSCLC. Direct comparisons of the efficacies of intrathecal methotrexate and pemetrexed are scarce. OS in our IM patients (median: 10.1 months) was better than the previously reported survival time of 3–8 months [16–18]; however, we only included eight IM patients, and further studies with larger sample sizes are needed. Although IC therapy is considered an effective treatment modality for LM patients, further studies are needed to identify the optimal agent for IC.

At present, there is still no consensus on the optimal frequency of administration. The frequency of administration in the current study was different from that in other studies [9, 19]. We had more frequent IC treatments in the first month (8 times in total). After patients develop devastating LM, IC is one of the few ways to control LM-related symptoms, especially when LM is resistant to targeted therapy or the patient has no operable mutation. Although the overall adverse reactions can still be controlled, we have not observed obvious benefits in clinical remission rate or OS. Considering that our study is retrospective, it is necessary to conduct prospective studies to further explore the efficacy of more frequent IC. In the future, we will explore the best dosage and concentration of intrathecal drugs through pharmacokinetic studies.

Most patients in the current study had *EGFR* mutations. All patients treated with EGFR-TKIs received third-generation agents (osimertinib or almonertinib), except for one patient who received second-generation agents for personal reasons. Sixteen patients started combined IC therapy after progression on third-generation EGFR-TKIs, and 8 patients received third-generation EGFR-TKIs after progression on first- or second-generation EGFR-TKIs. Given that most patients had received routine EGFR-TKIs before intrathecal therapy, no new mutations were found during intrathecal therapy, and LM-related symptoms developed at the same time, we considered that most of these LMs developed different degrees of EGFR-TKI resistance. We thus suggest that intrathecal therapy plays an important role in these patients with advanced LM. The OS in the current study was similar to that in a phase 1/2 clinical trial of IP after resistance to EGFR-TKIs (10.1 vs. 9 months) [9]. In the targeted therapy of LM, the concentration of EGFR-TKIs in CSF is often insufficient due to the blood‒brain barrier. Increased oral doses of EGFR-TKIs were used to overcome this deficiency. A retrospective study of 35 patients with LM from EGFR-mutated NSCLC who exhibited disease progression after failure of standard-dose EGFR-TKIs showed that high-dose erlotinib (various dosages and regimens of high-dose erlotinib were used: 200 mg on alternate days, 300 mg on alternate days, 300 mg every 3 days, 450 mg every 3 days, and 600 mg every 4 days) showed a radiologic response in 30% of patients and symptomatic improvement in neurologic symptoms in 50% of patients. The median survival time from the diagnosis of LM in patients treated with high-dose erlotinib and those not treated with erlotinib was not significantly different (6.2 months in the erlotinib arm vs. 5.9 in the control arm, P = 0.94) [20]. A multicentre phase I trial (BLOOM; NCT02228369) of 41 patients with LM from EGFR-mutated NSCLC who had disease progression on prior EGFR-TKI therapy showed an ORR of 62%, PFS of 8.6 months, and median OS of 11.0 months and reported CSF clearance in 11/40 patients (88) with 160 mg of osimertinib daily[21]. Park et al. carried out prospective research to assess the efficacy of 160 mg of osimertinib in CNS metastasis patients. They reported a 92.5% intracranial disease control rate and a 12.5% complete response rate in the LM cohort [22]. The AURA-LM analysis examined the clinical efficacy of 80 mg of osimertinib daily as a second-line treatment for EGFR T790M-NSCLC patients and demonstrated an ORR of 55%, CR of 27%, median PFS of 11.1, and median OS of 18.8 months [23]. At present, there is no comparative study on the efficacy of high-low-dose osimertinib in LM patients with NSCLC, nor has there been any study on whether increasing the dose can improve sensitivity after low-dose progression. Most of the patients included in our study had front-line osimertinib-resistant disease, and a dose increase was attempted in all of these patients. Some patients could not tolerate a high dose, and some patients did not exhibit relief of the symptoms of leptomeningeal metastasis after increasing the dose.

Both univariate and multivariate analyses showed that the use of bevacizumab was a good prognostic factor for NSCLC LM patients with combination therapy. Bevacizumab is a recombinant humanized IgG1 monoclonal antibody against vascular endothelial growth factor (VEGF), which specifically binds to VEGF to block its binding to its receptor, thus reducing angiogenesis, inducing the degeneration of existing blood vessels, inhibiting tumour formation, inhibiting immature angiogenesis, and inducing vascular normalization [24]. Animal studies have shown that antiangiogenic treatment can prolong the survival time of LM mice [25]. In our study, some patients who had already been administered bevacizumab before LM had stable disease at the primary lesion and other metastatic sites after LM progression, so bevacizumab was continued and combined with IC treatment. As an angiogenesis inhibitor against VEGFR, bevacizumab reduces brain edema and improves the blood‒brain barrier in patients with brain metastases and LM [24, 26]. We previously found that osimertinib combined with bevacizumab had a synergistic effect by modulating E-cadherin levels and increasing osimertinib levels in the brain, resulting in a significant difference in OS between LM patients treated with osimertinib combined with bevacizumab and osimertinib alone (p = 0.046) [27]. However, it is not clear whether bevacizumab has synergistic effects in patients with EGFR-TKI-resistant disease treated with IC. Because both osimertinib and bevacizumab can penetrate the blood‒brain barrier and have good efficacy in the central nervous system, the combination of bevacizumab with IC for the treatment of *EGFR*-mutated NSCLC LM warrants further in-depth study.

Our study had some limitations. First, this was a retrospective study. It was impossible to judge in 7 patients whether, before IC, they had NSCLC LM that was sensitive to subsequent targeted therapy because they had not received a third-generation EGFR-TKI and did not have next-generation sequencing results of cerebrospinal fluid. We believe that most patients with EGFR mutations had NSCLC LM that had become resistant to targeted therapy. However, a small number of patients may still have NSCLC LM that was sensitive to targeted therapy. Regarding the optimal timing of IC, further prospective studies are needed to determine if IC should be added when the patient is still sensitive to targeted therapy or after targeted therapy resistance. Second, the distribution of patients with IM and IP in our study was uneven, and the dosage and frequency of intrathecal injection were not uniform. Finally, the number of IM patients was small, and a larger sample size is needed to confirm the current results. Despite these limitations, this study provides important information regarding the treatment of LM patients with advanced NSCLC.

## Conclusions

This study retrospectively analysed the curative effect of IC in patients with refractory LM from NSCLC. The results suggested that combination therapies based on IC had a curative effect, were safe to use and may prolong patient survival. The use of bevacizumab is a good prognostic factor for NSCLC LM patients with combination therapy. Further prospective studies are needed to verify our conclusions and to explore the optimal dose, frequency, and treatment duration of intrathecal pemetrexed and methotrexate administration in patients with LM.

## Electronic supplementary material

Below is the link to the electronic supplementary material.

Supplementary Material 1

Supplementary Material 2

## Data Availability

The datasets used and/or analysed during the current study are available from the corresponding author on reasonable request.
