# Supplementary material for: Intrathecal chemotherapy combined with systemic therapy in patients with refractory leptomeningeal metastasis of non-small cell lung cancer: a retrospective study
Source: BMC Cancer. 2023 Apr 11;23:333. doi: 10.1186/s12885-023-10806-5 (PMC10088274; doi:10.1186/s12885-023-10806-5)
Supplement: Supplementary file 1 — Supplementary Material 1 [file 12885_2023_10806_MOESM1_ESM.docx]

| Supplementary Table 1 | | | | | | | | | | | |
| --- | --- | --- | --- | --- | --- | --- | --- | --- | --- | --- | --- |
| Patient | Max dose of IC | | No. of IC | Patient | Max dose of IC | | No. of IC | Patient | Max dose of IC | | No. of IC |
| 1 | | 12mg | 5 | 17 | | 30mg | 13 | 33 | | 10mg | 11 |
| 2 | | 20mg | 9 | 18 | | 20mg | 22 | 34 | | 20mg | 3 |
| 3 | | 20mg | 5 | 19 | | 20mg | 4 | 35 | | 10mg | 20 |
| 4 | | 40mg | 7 | 20 | | 12mg | 4 | 36 | | 40mg | 19 |
| 5 | | 20mg | 8 | 21 | | 20mg | 8 | 37 | | 30mg | 12 |
| 6 | | 12mg | 5 | 22 | | 40mg | 5 | 38 | | 20mg | 3 |
| 7 | | 12mg | 6 | 23 | | 15mg | 3 | 39 | | 30mg | 8 |
| 8 | | 12mg | 8 | 24 | | 50mg | 3 | 40 | | 30mg | 11 |
| 9 | | 12mg | 7 | 25 | | 20mg | 9 | 41 | | 30mg | 5 |
| 10 | | 30mg | 12 | 26 | | 20mg | 2 |  | |  |  |
| 11 | | 20mg | 7 | 27 | | 10mg | 9 |  | |  |  |
| 12 | | 20mg | 12 | 28 | | 20mg | 2 |  | |  |  |
| 13 | | 20mg | 11 | 29 | | 20mg | 9 |  | |  |  |
| 14 | | 20mg | 7 | 30 | | 30mg | 3 |  | |  |  |
| 15 | | 12mg | 2 | 31 | | 20mg | 3 |  | |  |  |
| 16 | | 10mg | 4 | 32 | | 30mg | 18 |  | |  |  |
| IC, intrathecal chemotherapy; | | | | | | | | | | | |
