# Supplementary figures and images for: Intrathecal chemotherapy combined with systemic therapy in patients with refractory leptomeningeal metastasis of non-small cell lung cancer: a retrospective study
Source: BMC Cancer. 2023 Apr 11;23:333. doi: 10.1186/s12885-023-10806-5 (PMC10088274; doi:10.1186/s12885-023-10806-5)

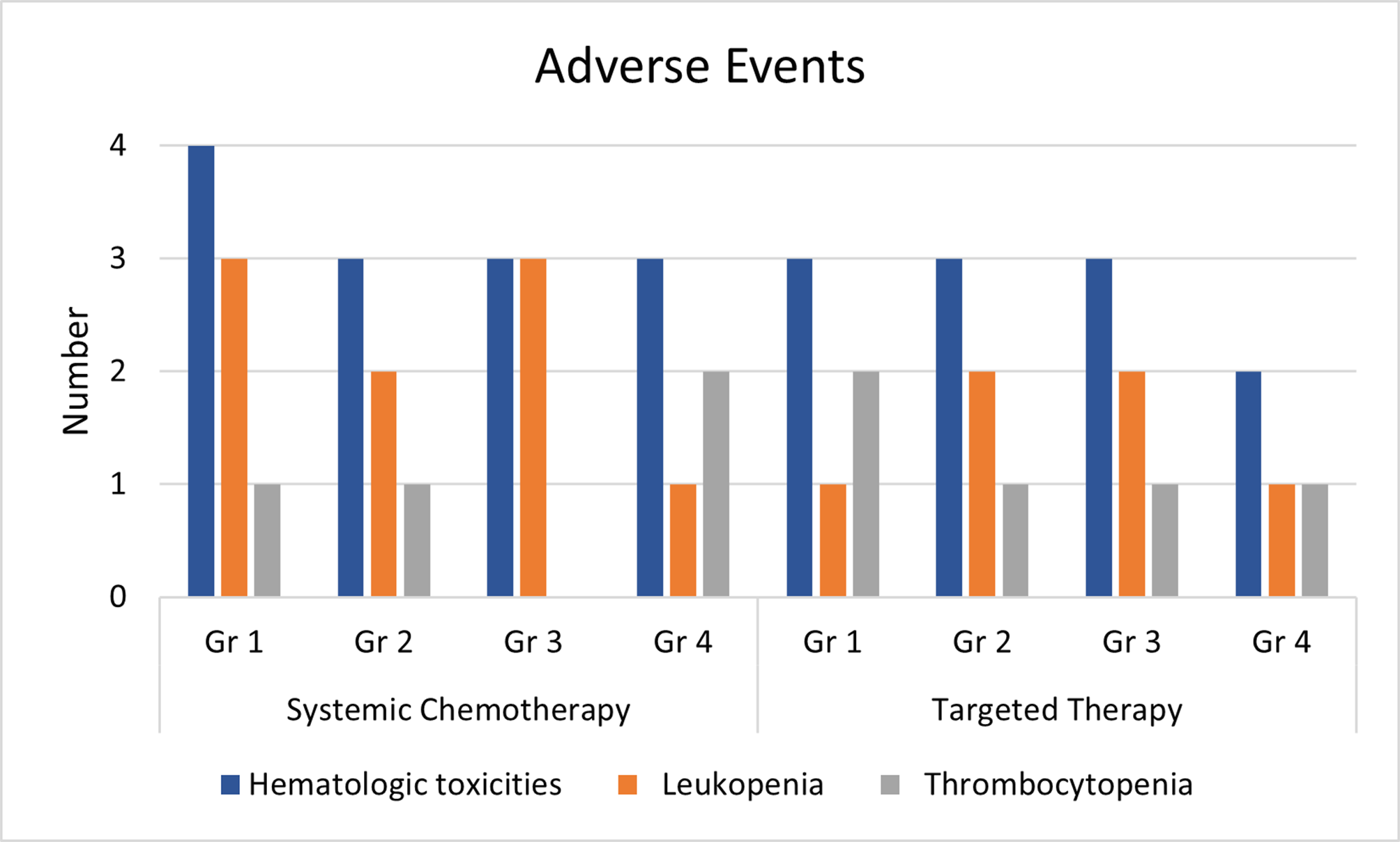

Supplement: Supplementary file 2 — Supplementary Material 2 [file 12885_2023_10806_MOESM2_ESM.tif]
